# Biologically Active α-Amino Amide Analogs and γδ T Cells—A Unique Anticancer Approach for Leukemia

**DOI:** 10.3389/fonc.2021.706586

**Published:** 2021-07-12

**Authors:** Ahmed Al Otaibi, Subuhi Sherwani, Salma Ahmed Al-Zahrani, Eida Mohammed Alshammari, Wahid Ali Khan, Abdulmohsen Khalaf D. Alsukaibi, Shahper Nazeer Khan, Mohd Wajid Ali Khan

**Affiliations:** ^1^ Department of Chemistry, College of Sciences, University of Ha’il, Ha’il, Saudi Arabia; ^2^ Department of Biology, College of Sciences, University of Ha’il, Ha’il, Saudi Arabia; ^3^ Department of Clinical Biochemistry, College of Medicine, King Khalid University, Abha, Saudi Arabia; ^4^ Interdisciplinary Nanotechnology Centre, Aligarh Muslim University, Aligarh, India; ^5^ Molecular Diagnostic and Personalised Therapeutics Unit, University of Ha’il, Ha’il, Saudi Arabia

**Keywords:** cancer, biologically active molecules, γδ T cells, cytotoxicity, combinational therapy, leukemia

## Abstract

Advanced stage cancers are aggressive and difficult to treat with mono-therapeutics, substantially decreasing patient survival rates. Hence, there is an urgent need to develop unique therapeutic approaches to treat cancer with superior potency and efficacy. This study investigates a new approach to develop a potent combinational therapy to treat advanced stage leukemia. Biologically active α-amino amide analogs (RS)-N-(2-(cyclohexylamino)-2-oxo-1-phenylethyl)-N-phenylpropiolamide (α-AAA-A) and (RS)-N-(2-(cyclohexylamino)-2-oxo-1-phenylethyl)-N-phenylbut2-enamide (α-AAA-B) were synthesized using linear Ugi multicomponent reaction. Cytotoxicities and IC_50_ values of α-AAA-A and α-AAA-B against leukemia cancer cell lines (HL-60 and K562) were analyzed though MTT assay. Cytotoxic assay analyzed percent killing of leukemia cell lines due to the effect of γδ T cells alone or in combination with α-AAA-A or α-AAA-B. Synthesized biologically active molecule α-AAA-A exhibited increased cytotoxicity of HL-60 (54%) and K562 (44%) compared with α-AAA-B (44% and 36% respectively). Similarly, α-AAA-A showed low IC_50_ values for HL-60 (1.61 ± 0.11 μM) and K562 (3.01 ± 0.14 μM) compared to α-AAA-B (3.12 ± 0.15 μM and 6.21 ± 0.17 μM respectively). Additive effect of amide analogs and γδ T cells showed significantly high leukemia cancer cell killing as compared to γδ T cells alone. A unique combinational therapy with γδ T cells and biologically active anti-cancer molecules (α-AAA-A/B), concomitantly may be a promising cancer therapy.

## Introduction

Cancer is a disease characterized by uncontrolled growth of cells, causing mortality worldwide. In 2018, it was estimated that cancer was the second leading cause of death worldwide and was responsible for 9.6 million deaths (1). In 2020, an estimated 1.8 million new cancer cases were diagnosed, and 606,520 cancer related deaths occurred in the US (2). According to the World Health Organization, the statistics for Saudi Arabia reported that, of the total deaths in the year of 2012, 10.2% deaths were due to cancer. A substantial number of cancer related deaths in the Saudi adult population were due to colorectal cancer (males; 19.3%) and breast cancer (females; 29%) [World Health Organization-Cancer Country Profiles, (3)]. According to statistics reported in 2018, the most common type of cancer among Saudi children of both sexes was leukemia (34.6%) (4). There are a number of conventional drugs available for the treatment of leukemia. However, severe toxicities (cardiotoxicity, neuropathy, hepatotoxicity, renal toxicity etc.) have been registered for almost all the drugs, which may also cause morbidity and mortality in patients (5–8). Best approach to reduce the burden of these toxicities is to strategize for better outcome for patients.

Cancer treatment options may include the use of various techniques ranging from surgery, radiation, medications and/or other therapies to cure, shrink or stop the progression of a cancer. Monotherapeutic treatments have limitations when it comes to advanced stage cancers, due to disease progression which makes the disease more complex (9). Scientists and clinicians are making concerted efforts to develop pharmacological and immunological interventions for multiple targets, which are efficient, cost effective and could potentially increase the life span of patients with advanced stage cancers. Some clinical studies on pediatric leukemia showed that the efficacy of monotherapies was remarkably enhanced when another drug was administered together during the treatment (10). Currently, biologically active molecules and adoptive cell therapies are considered to be the most advanced areas of research in the development of potential therapeutics for cancer treatments.

Some important molecules, such as Brentuximab Vedotin, Gemtuzumab Ozogamicin, Ado-trastuzumab emtansine, polatuzumab vedotin-piiq, and inotuzumab ozogamicin, are combined with monoclonal antibodies specific to surface antigens present on particular tumor cells and are used as combinational-targeted cancer therapies (11). There are a number of newly synthesized α-amino amide derivatives, which show potent anticancer and cytotoxic activities against a wide range of cancer cell lines (12). Derivatives of 3-cyano pyridine also exhibited cytotoxic activities against many human MCF-7, HCT-116, and HepG-2 cancer cell lines (13). However, the cytotoxic effects of these biologically active anticancer molecules vary depending on the type of cancer, as well as dosage. Adaptive cancer therapy is involved in the eradication of tumor cells.

Various immune cells (T cells, NK cells, dendritic cells etc.) work differently in immunotherapies for cancer. γδ T cells are one of the unconventional T cells, which can be distinguished from αβ T cells (major T cell subset). These cells express Vγ9Vδ2-TCR on their cell surface. Other than tumor killing, γδ T cells have numerous important functions in immunity, including cytokine production and mobilization of other types of immune cells (at least *in vitro*), which favor these cells as an anticancer therapy option (14–16). γδ T cells led clinical trials have used these as effector cells in the treatment of various cancers, including breast carcinoma (17), colorectal carcinoma (18) and renal cell carcinoma (19) and found them to be well tolerated. Even *in vivo* infusion of γδ T cells recognized tumor cells and showed cytotoxicity against them (20).

The aims of this study are to elucidate the role of α-amino amide analogs (α-AAA) (RS)-N-(2-(Cyclohexylamino)-2-oxo-1-phenylethyl)-N-phenylpropiolamide (α-AAA-A) and (RS)-N-(2-(Cyclohexylamino)-2-oxo-1-phenylethyl)-N-phenylbut2-enamide (α-AAA-B), as anticancer agents and investigate their anti-proliferative or anti-metastatic activities alone or in combination with γδ T cells against leukemia cancer cell lines HL-60 and K562.

## Materials and Methods

RPMI-1640 medium, lymphoprep^™^, 0.9% saline (NaCl), millipore filter (0.22 μM), fetus calf serum (FCS), L-glutamine (200 mM; 100×), pen/strep (10,000 unit/ml pen and 10,000 units/ml strep), MEM sodium pyruvate (100 mM), non-essential amino acids (10 mM; 100×). IL-2 (100 IU/ml) were from GIBCO Life Technologies, (USA). Human recombinant interleukin-2 and interleukin-15 were from Novartis (Switzerland) and Miltenyi Biotec (Germany), respectively. Mouse monoclonal antibodies specific for CD3 (UCHT1), TCR-Vγ9 (Immu360) were from Beckman Coulter (USA). Fixable aqua dead cell stain kit was from Invitrogen-Life Technologies (USA). Human IgG, methanol, aniline, benzaldehyde, propiolic acid, cyclohexyl isocyanide, dimethyl sulfoxide (DMSO) and 3-[4,5-dimethylthiazol-2-yl]-2,5-diphenyltratrazolium bromide were purchased from Sigma Aldrich (USA). Zoledronic acid injection (4 mg) were purchased from Cipla, (India). HL-60 and K562 cell lines were received from ATCC (USA).

### Synthesis of Biologically Active Molecules

Biologically active α-amino amide analogs (RS)-N-(2-(Cyclohexylamino)-2-oxo-1-phenylethyl)-N-phenylpropiolamide (α-AAA-A) and (RS)-N-(2-(Cyclohexylamino)-2-oxo-1-phenylethyl)-N-phenylbut2-enamide (α-AAA-B) were synthesized using linear Ugi multicomponent reaction (batch reaction with methanol) as published previously (12). Briefly, for the synthesis of α-AAA-A, a solution was prepared using methanol (5 ml), aniline (0.09 ml; 1 mmol) and benzaldehyde (0.1 ml; 1 mmol). This solution was stirred at 25°C for half an hour. Then propiolic acid (0.06 ml; 1 mmol) was added to this solution, followed by the addition of cyclohexyl isocyanide (0.11 ml; 1 mmol). This reaction mixture was further stirred at 25°C for 24 h till a precipitate was formed, which was washed with diethyl ether and dried to obtain a white solid (0.2 gm).

For the synthesis of α-AAA-B, similar linear Ugi multicomponent reaction was used. A solution was prepared using methanol (5 ml), aniline (0.09 ml; 1 mmol) and benzaldehyde (0.1 ml; 1 mmol). This solution was stirred at 25°C for half an hour. Then 2-butyric acid (23 gm; 1 mmol) was added to this solution, followed by the addition of cyclohexyl isocyanide (0.11 ml; 1 mmol). This reaction mixture was stirred further at 25°C for 24 h till a precipitate formed, which was washed with diethyl ether and dried to obtain a white solid (0.21 gm).

### Cancer Cell Lines

HL-60 and K562 were grown in complete RPMI 1640 medium which included nonessential amino acids and was supplemented with 10% FCS, 1 mM sodium pyruvate and 2 mM L-glutamate. All cells were grown at 37°C in 95% air with the addition of 5% CO_2_.

### MTT Assay

The 3-[4,5-dimethylthiazol-2-yl]-2,5-diphenyltratrazolium bromide (MTT) assay has been done as described previously (21, 22) with slight modifications. Briefly, the incubation of 1 × 10^5^ cells/mL cancer cells (HL-60 or K562) in complete RPMI medium, with or without the addition of amide analogs (α-AAA-A or α-AAA-B), was followed by incubation for different durations (4–24 h) at 5% CO_2_ and 37°C. Thereafter, the cells were treated with 100 μl of MTT (5 mg/ml). Four hours later, the entire medium, including MTT solution, was aspirated from the wells. The remaining formazan crystals were dissolved in DMSO (50 μl) and absorbance was measured at 570 nm using a 96 well microplate reader. The cytotoxicity index was determined using the untreated cells as negative control. The percentage of cytotoxicity was calculated using the background-corrected absorbance as follows:

$$\%\;\text{cytotoxicity}=\frac{(1\;-\text{absorbance}\;\text{of}\;\text{experimental well})\times 100}{\text{absorbance of negative control well}}$$

### γδ T Cell Lines

γδ T cell lines were prepared as described in our previous research publication (14). Briefly, γδ T cells which were present in freshly isolated PBMCs, were stimulated using 1 µM zoledronate and were cultured in complete RPMI 1640 medium at a density of 10^6^ cells/ml in 24-well culture plate and kept in CO_2_ (5%) incubator at 37°C for 2 weeks. The level of water in the incubator was checked often to prevent decrease in levels of medium. Cytokines IL-2 (100 IU/ml) and IL-15 (10 ng/ml) were added to the culture at day 3, 6, 8 and 11, and cells were split and fresh medium added. At day 14, the percent γδ T cells in culture were examined by flow cytometry. Fixable aqua dead cell stain kit was used to detect percent live γδ T cells. Total number of live cells was counted using trypan blue stain.

### Cell Surface Staining

Approximately 100,000 to 500,000 PBMCs, before and after expansion, were plated in a total volume of up to 250 μl. These were centrifuged at 1100 rpm for 3 min at 4°C in a centrifuge. The cell pellets were then washed with 200 μl phosphate buffer saline (PBS). Three microliters of fixable aqua dead cell stain were added and incubated for 15 min in the dark, followed by the addition of 200 μl PBS containing 2% FCS (FACS buffer). After incubation, cells were centrifuged (1100 rpm for 3 mins) and the supernatant discarded. Cells were blocked with 100 μl of 1:1000 diluted human IgG for 15 min on ice. After incubation cells were centrifuged (1100 rpm for 3 mins) and pelleted. Again, the supernatant was discarded. Then cells were stained with 40 μl cocktails of antibodies & isotype by further incubating for 20 min on ice in the dark. Approximately 160 μl FACS buffer was added after incubation and then the mixture was centrifuged (1100 rpm for 3 min) again. Finally, cells were resuspended in 100 μl FACS buffer and were analyzed by flow cytometer (Beckman Coulter Flow cytometer, Navios A52101).

### Cytotoxic Effect of γδ T Cells

Tumor cell-killing activity of expanded γδ T cell (14 days) was tested on the leukemia cancer cell lines (HL-60 and K562). Cancer cells were labeled with the membrane dye CellVue. Labeled cancer cells were incubated together with γδ T cells at increasing target cell (T) to effector (E) ratios (1:1; 1:5; 1:10; 1:25) for 12 h in CO_2_ (5%) incubator at 37°C. Cancer cell-killing was determined by flow cytometry.

### Combinational Cytotoxic Effect

Combinational cancer cell killing was determined by flow cytometry. Cancer cells were labeled with the membrane dye CellVue. Labeled cancer cells were incubated together with γδ T cells at increasing target cell (T)/effector (E) ratios (1:1; 1:5; 1:10; 1:25) for 12 h in CO_2_ (5%) incubator at 37°C. Combinational cytotoxic effects of biologically active molecules (α-AAA-A and α-AAA-B) and expanded γδ T were analyzed for leukemia cancer cell lines (HL-60 and K562). α-AAA-A or α-AAA-B were added to the culture with different target (HL-60 or K652)/effector (γδ T cells) ratios. Cancer cells and γδ T cells alone served as controls (ctrl). For HL-60 cancer cell line, the concentrations used of α-AAA-A and α-AAA-B were 3.125 and 6.26 μM, respectively. For K-562 cancer cell line, concentrations used of α-AAA-A and α-AAA-B were 6.26 and 12.5 μM, respectively. Each assay is representative of three experiments.

### Statistical Analysis

All results were expressed as the mean ± SD. Multiple comparisons between data were done using software OriginPro 8.5 followed by Student’s t test. The p value for significance was set at < 0.05.

## Results

**Figure 1:**
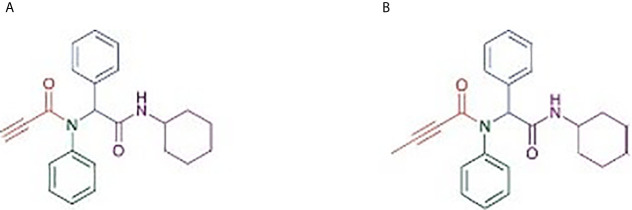
Structure of α-amino amide analogs (RS)-N-(2-(Cyclohexylamino)-2-oxo-1-phenylethyl)-N-phenylpropiolamide **(A)** and (RS)-N-(2-(Cyclohexylamino)-2-oxo-1-phenylethyl)-N-phenylbut2-enamide **(B)**, synthesized using linear Ugi multicomponent reaction (batch reaction with methanol).

Most of the drugs used in the treatment of leukemia cause severe toxicities and, hence, it is important to synthesize and test novel biologically active molecules which can potentially be used as anti-cancer agents with low toxicities. Two biologically active compounds (**Figure 1**), α-AAA-A and α-AAA-B, were synthesized using linear Ugi multicomponent reaction (batch reaction with methanol). Both molecules were isolated without any further purification and were stable at room temperature. The cytotoxic effects of both biologically active molecules were analyzed for two different leukemia cancer cell lines (HL-60 and K562).

### Cytotoxic Effect of Biologically Active Molecules

**Figure 2:**
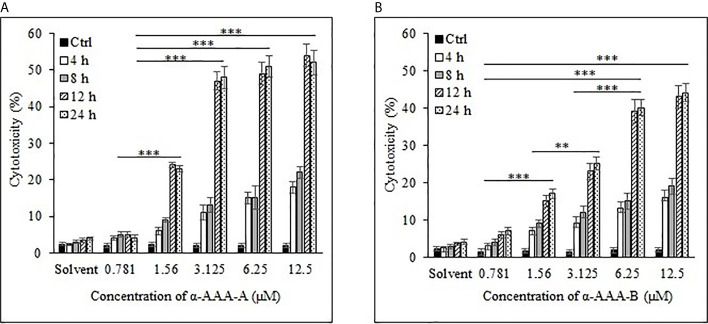
Cancer cell line HL-60 cytotoxicity by α-AAA-A **(A)** and α-AAA-B **(B)** at different concentrations and durations of incubations. ***P < 0.001, when comparing cytotoxicity by α-AAA-A at 12.5, 6.25 and 3.125 μM with 1.56 μM after 12 and 24 h of incubations. ***P < 0.001, on comparing cytotoxicity by α-AAA-B at 12.5, 6.25 and 3.125 μM with 1.56 μM after 12 and 24 h of incubations. **P < 0.01, on comparing cytotoxicity by α-AAA-B at 1.56 with 3.125 μM. ***P < 0.001, on comparing cytotoxicity by α-AAA-A and α-AAA-B at 0.781 and 1.56 μM. Cancer cells without molecules served as control. Solvent was also used for different conditions.

Cancer cell cytotoxicity was measured by MTT assay, and the absorbance was recorded at 570 nm. Comparably high cytotoxicity of HL-60 and K562 was recorded by α-AAA-A as compared to α-AAA-B. HL-60 cells visibly showed remarkably high inhibition at 3.125 μM after 12 h of incubation period, when co-cultured with α-AAA-A (**Figure 2A**). However, with α-AAA-B, increased inhibition was achieved at concentration of 6.25 μM after 12 h of incubation (**Figure 2B**). Highest percent of HL-60 cytotoxicity was 54% with α-AAA-A and 44% with α-AAA-B, respectively. No significant differences were observed in percent cell inhibition of HL-60 cells among α-AAA-A concentrations of 3.125, 6.25, and 12.5 μM for 12 and 24 h. However, significant differences (*p <* 0.001) were observed in percent cell inhibition of HL-60 cells between 1.56 and 3.125 μM concentrations after 12 and 24 h of incubations (**Figure 2A**). Similar patterns of cytotoxicity were observed with α-AAA-B, with exception of low percent inhibition at 3.125 μM (**Figure 2B**). Cancer cell cytotoxicities at 0.781 μM were significantly low for both molecules when compared with other concentrations.

**Figure 3:**
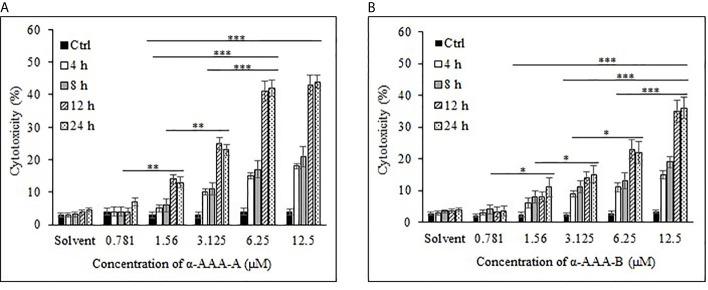
Cancer cell line K562 cytotoxicity by α-AAA-A **(A)** and α-AAA-B **(B)** at different concentrations and time durations of incubations. ***P < 0.001, on comparing cytotoxicity by α-AAA-A at 12.5, 6.25 and 3.125 μM with 1.56 μM after 12 and 24 h of incubations. ***P < 0.001, on comparing cytotoxicity by α-AAA-B at 12.5 with 6.25 μM; 12.5 with 3.125 μM; 12.5 with 1.56 μM after 12 and 24 h of incubations. **P < 0.01, on comparing cytotoxicity by α-AAA-A at 3.125 with 1.56 μM; 1.56 with 0.781 μM. *P < 0.05, on comparing cytotoxicity by α-AAA-B at 6.25 with 3.125 μM; 3.125 with 1.56 μM; 1.56 with 0.781 μM.

Cancer cell line K562 cells showed high cytotoxicity at higher concentrations of 6.25 and 12.5 μM when incubated with α-AAA-A (**Figure 3A**) and α-AAA-B (**Figure 3B**), respectively post 12 h incubation periods. Highest percent of K562 cell cytotoxicities were 44% with α-AAA-A and 36% with α-AAA-B. Differences in percent cytotoxicity of K562 cells, using α-AAA-A at concentrations of 6.25 and 12.5 μM for 12 and 24 h, were not significant. However, observed percent cell cytotoxicity using α-AAA-A at concentrations of 0.781, 1.56, and 3.125 μM, were significantly lower (*p <* 0.001) when compared with concentrations 6.25 and 12.5 μM (**Figure 3A**).

For the molecule α-AAA-B, percent cytotoxicity of K562 cells at concentrations of 0.781, 1.56, 3.125, and 6.25 μM were significantly lower (*p* < 0.001) when compared with 12.5 μM (**Figure 3B**).

To determine the effect of solvent of molecules on the MTT assays, solvent in absence of molecules was also used as control, and as expected, it did not show any appreciable cytotoxicity with different durations of incubation for both cancer cell lines (**Figures 2** and **3**).

These results show that α-AAA-A exhibited more cytotoxic effects on both leukemia cancer cell lines as compared to α-AAA-B. However, leukemia cancer cell line K562 showed more resistance against both molecules. Conventional FDA-approved drug methotrexate was also used to detect cytotoxicities against both leukemia cancer cell lines HL-60 and K562 (**Supplementary Figure 1**). Appreciable cytotoxicities were observed with higher concentrations of methotrexate.

**Figure 4:**
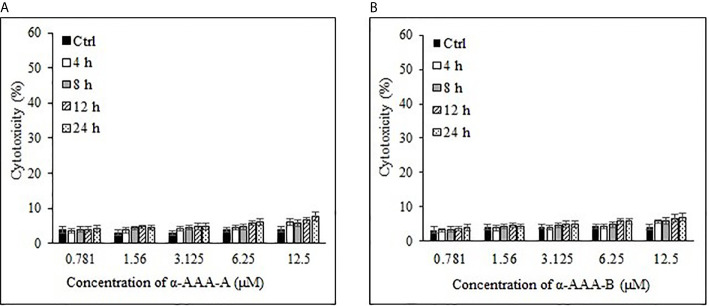
Cytotoxicities of freshly isolated PBMCs cytotoxicities by α-AAA-A **(A)** and α-AAA-B **(B)** at different concentrations and time durations of incubation. PBMCs without molecules served as control.

**Figure 5:**
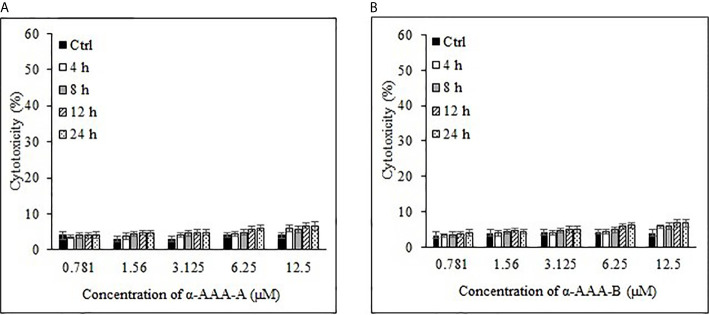
Analysis of normal breast cell line MCF10A cytotoxicity by α-AAA-A **(A)** and α-AAA-B **(B)** at different concentrations and time durations of incubation. Control means MCF10A cells without molecules served as control.

Freshly isolated PBMCs were used as negative controls to test the cytotoxic effects of both analogs α-AAA-A (**Figure 4A**) and α-AAA-B (**Figure 4B**) using MTT assay. At varying concentrations (0.781–12.5 μM) of α-AAA-A and α-AAA-B, low cytotoxicities (4–7.6%) were observed with both the analogs. Similar findings were observed for another negative control used, i.e., normal breast cell line MCF10A, in which cytotoxicities were evaluated against both molecules α-AAA-A (**Figure 5A**) and α-AAA-B (**Figure 5B**). The cytotoxicity levels were found to be in the range of (3.3–7.1%) with the analogs.

### IC_50_ Estimation

**Figure 6:**
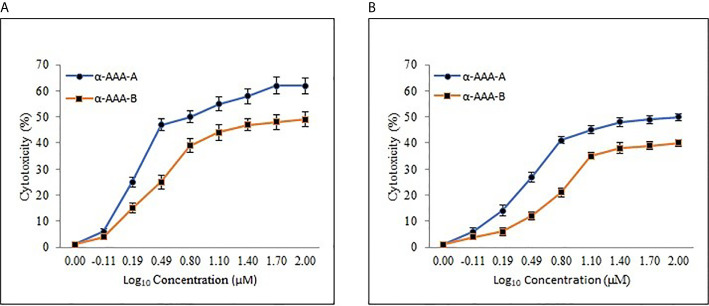
Dose-response curve of α-AAA-A and α-AAA-B added to the HL-60 **(A)** and K562 **(B)** cancer cell culture. The incubation period for all the assays was 12 h. The IC_50_ was calculated from the curve generated. The lower the IC_50_, the more cytotoxic the molecule is to specific the respective cancer cell line.

The half-maximal inhibitory concentration (IC_50_) values were extrapolated from the concentration-response log_10_ graphs, using MTT assay. The IC_50_ values were calculated for HL-60 (**Figure 6A**) and were achieved at 1.61 ± 0.11 μM and 3.12 ± 0.15 μM for α-AAA-A and α-AAA-B, respectively. Cells of the leukemia cell line K562 (**Figure 6B**) exhibited the IC_50_ values at 3.01 ± 0.14 μM and 6.21 ± 0.17 μM for α-AAA-A and α-AAA-B, respectively. These results showed α-AAA-A showed greater cytotoxic effect as compared with α-AAA-B for both leukemia cancer cell lines HL-60 and K562.

Cytotoxic effect was demonstrated by both analogs in leukemia cancer cell lines HL-60 and K562, which varied with the concentrations of the molecules. Hence, both α-amino amide analogs can exhibit cytotoxicity toward both cancer cell lines in a dose- and time-dependent manner.

### Expansion of Human γδ T Cells

Freshly isolated PBMCs (10^6^ cells/ml) were stimulated using 1 µM zoledronate and were cultured in complete RPMI 1640 medium. Cytokines IL-2 (100 IU/ml) and IL-15 (10 ng/ml) were added to the culture according to the protocol using fresh medium. After fourteen days of culture, high yield (2279.2 ± 487) of pure γδ T (90.7 ± 4.6%) cells was recovered (**Table 1**). The purity and viability of expanded γδT cells were examined by flow cytometry.

**Table 1:** *In vitro* expanded of γδ T cells from freshly isolated PBMCs in response to zoledronate (1 µM).

Donors	4
PBMC	1 × 10^6^
γδ-T cells at day 0	0.019 ± 0.007 × 10^6^
After 14 days expansion	
% Live Cells	85.9 ± 4.7
% CD3^+^ cells	95.5 ± 2.8
% γδ-T cells (%)	90.7 ± 4.6
Total number of cells	58.2 ± 6.1 × 10^6^
*Number of γδ-T cells	43.3 ± 8.5 × 10^6^
^#^Expansion fold	2279.2 ± 487

Each donor PBMCs were cultured in triplicates.

Each value is the mean ± SD from 4 different donors.

*Total γδ-T cells (day 14) were calculated as: total cells × total live cells (%) × CD3+ cells (%) × γδ-T cells (%).

^#^Expansion folds were calculated as: total γδ T cells (day 14)/γδ-T (day 0).

**Figure 7:**
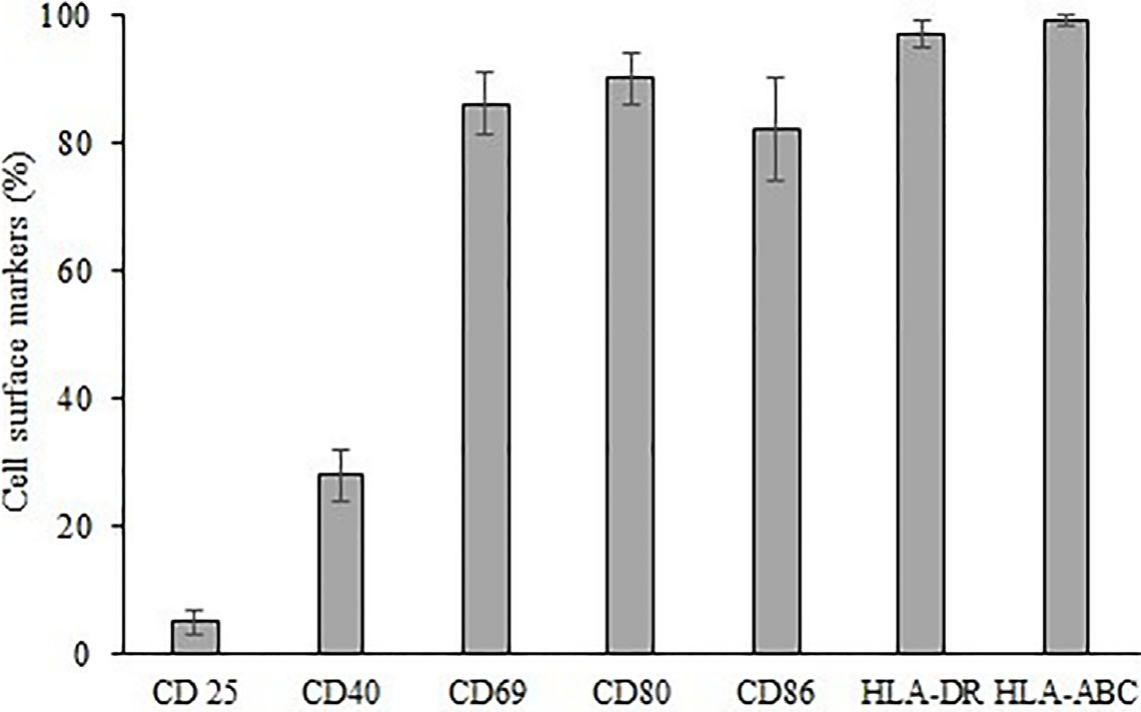
Expression of cell surface markers CD 25, CD40, CD69, CD80, CD86, HLA-DR, HLA-ABC on expanded γδ T cells.

These expanded γδ T cells were also analyzed for activation and costimulatory cell surface molecules (**Figure 7**). Expanded cells express high percent of activation molecule CD69 (86%), costimulatory molecules CD40, CD80, and CD86 (19%, 90% and 82% respectively). Moreover, high percent of major histocompatibility molecules HLA-DR and HLA-ABC were also expressed (97% and 99% respectively). CD25 a proliferation marker is expressed in an exceptionally small (5%) population of expanded cells.

### Cytotoxic Effect of γδ T Cells

**Figure 8:**
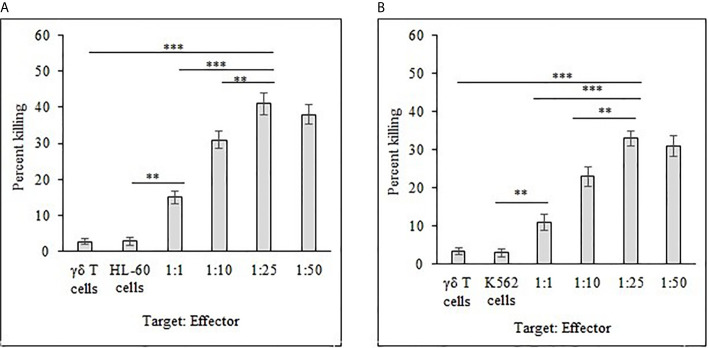
Percent killing of HL-60 **(A)** and K562 **(B)** cancer cells by 14 day expanded γδ T cells. Different T:E ratios were incubated together for a duration of 12 h. γδ T cells and leukemia cancer cells (HL-60 and K562) alone served as controls. For both HL-60 and K562, ***P < 0.001 on comparison of percent cell killing at the T:E ratios of 1:25 to 1:1. **P < 0.01 on comparison of percent cell killing at the T:E ratios of 1:25 to 1:10.

Freshly expanded γδ T cells showed cytotoxicity toward HL-60 (**Figure 8A**) and K562 (**Figure 8B**) cancer cells analyzed by flow cytometer. There was significant increase (*P* < 0.001) in killing of both HL-60 and K562 cells at the target (T) and effector (E) ratios of 1:10, 1:25 and 1:50 as compared to 1:1 T:E ratio. However, there are also significant differences (*P* < 0.01) between both cell lines in percent cell killing on comparison of T:E ratios of 1:10 and 1:25. Highest tumor cell killing for HL-60 cells was evident at a T:E ratio of 1:25 (41%). Similarly, highest killing for K562 cells was achieved at a T:E ratio of 1:25 (33%). It is evident that lesser numbers of γδ T cells (1:10, T:E ratio) are not able to successfully kill tumor cells. Also, γδ T cells kill HL-60 cells more effectively as compared with K562 cells. γδ T cells as well as both leukemia cancer cell lines (HL-60 and K562), when present alone, did not show substantial cell killing after 12 h of incubation under similar culture conditions.

### Combinational Cytotoxic Effect

**Figure 9:**
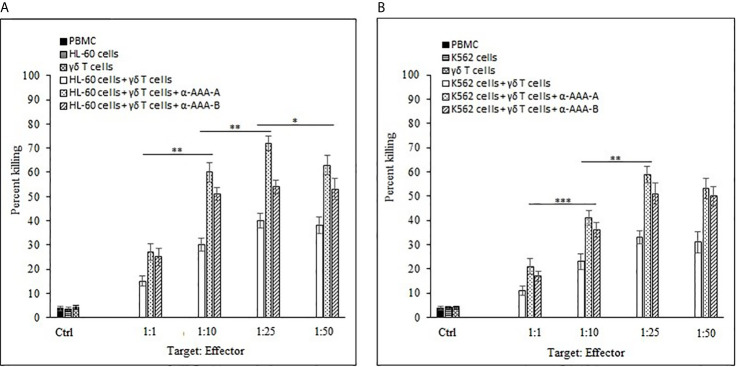
Combinational effect of both amide analogs and expanded γδ T cells. Percent killing of HL-60 **(A)** and K562 **(B)** cancer cells by 14 days expanded γδ T cells in combination with α-AAA-A or α-AAA-B. Different T:E ratios were incubated together for a duration of 12 h. PBMCs alone, cancer cells alone and γδ T cells alone served as controls (ctrl). For HL-60 cancer cell line **(A)**, the concentrations of α-AAA-A and α-AAA-B used were 3.125 and 6.26 μM, respectively. For K-562 cancer cell line **(B)**, concentrations of α-AAA-A and α-AAA-B used were 6.26 and 12.5 μM, respectively. Significance represented as ***P < *0.001*, **P < 0.01 and *P < 0.05.

The combinational effects of both synthesized analogs (α-AAA-A and α-AAA-B) and expanded γδ T cells were tested *in vitro* for their cytotoxic effects on leukemia cancer cell lines (HL-60 and K562) using flow cytometer. Significantly high percent of cancer cell killing was observed when these cell lines were co-cultured with molecule (α-AAA-A or α-AAA-B) and γδ T cells together. Highest percent of killings of HL-60 cells (72%) (**Figure 9A**) and K562 cells (59%) (**Figure 9B**) were observed in culture conditions which contained α-AAA-A and γδ T cells at 1:25 target to effector ratio. PBMCs alone, cancer cells alone and γδ T cells alone served as controls. These results suggested that α-AAA-A, in combination with γδ T cells, exhibited better leukemia cancer cell killing as compared to α-AAA-B and γδ T cells alone.

The control experiments were designed for co-culture of expanded γδ T cells with both molecules (α-AAA-A and α-AAA-B) at various concentrations to analyze the effect of α-amino amide analogs on 14 days expanded cells. Results showed no substantial cytotoxic effects (≤ 4%) on these cells (**Supplementary Figure 2**). One of the conventional drugs for leukemia, “methotrexate,” when tested together with γδ T cells, showed appreciable cytotoxicity (**Supplementary Figure 3**).

## Discussion

For the development of potential cancer treatments, strategies need to focus on the inhibition of proliferative potential and migration of cancer cells, as well as destruction of tumors. Various available molecular drugs for the treatment of cancers can causes high toxicity and are often not well tolerated (23). In leukemia patients, toxicities such as cardiotoxicity, neuropathy, hepatotoxicity, renal toxicity, and so on, might be potential reasons for morbidity and mortality in these patients (5–8). Best approach to reduce the burden of these toxicities is to devise strategies for better patient outcomes.

In addition to palliative care, personalized cancer therapy options need to be investigated, especially in the case of advanced stage cancer patients, often characterized by a higher death rate. In such cases, it is challenging to treat cancers with monotherapies. To overcome these challenges and enhance efficacy, therapies directed at different signaling pathways or an amalgamation of different targeted therapies are needed. Hence combinational therapeutics may be a more effective route for a durable tumor response. Combinational therapies can act simultaneously, targeting different pathways to inhibit not only tumor cell proliferation potential, but also tumor cell killing (9, 24–26). Two of the most advanced research areas involved in the field of oncotherapeutics, i.e., medicinal chemistry and adoptive cell therapy, are currently involved in development of potential anti-cancer therapeutics. Hence, in this study, we have tested and evaluated two therapies and their additive effects *in vitro*.

Biologically active molecules have inherent advantages over adaptive immunotherapies, as these molecules can reach a wider spectrum of molecular targets, including intracellular targets and even those present deep in the tumor milieu (27). The anticancer potency of two α-amino amide analogs (RS)-N-(2-(cyclohexylamino)-2-oxo-1-phenylethyl)-N-phenylpropiolamide and (RS)-N-(2-(cyclohexylamino)-2-oxo-1-phenylethyl)-N-phenylbut2-enamide, which were synthesized using linear Ugi multicomponent reaction, was tested for leukemia cancer cell lines. These molecules are stable at room temperature and cost effective (12). It has been previously evaluated that amide derivatives exhibit effective anticancer properties against various cancer cell lines, such as breast cancer (MCF-7 and MDA-MB-231), lung cancer (A549), and prostate cancer (DU-145) (28). Similarly, our synthesized analogs (α-AAA-A and α-AAA-B) showed cytotoxic effects against HL-60 and K562 cancer cells, which varied depending on concentrations of the molecules used. Notably, the IC_50_ value of α-AAA-A is far less as compared with α-AAA-B for both cancer cell lines. In a previous study, it has been observed that analog A also showed low IC_50_ as compared to analog B, when tested for other cancer cell lines such as HT29, U87, A2780, H680, A431, Du145 etc., suggesting better efficiency of α-AAA-A as an anticancer molecule in comparison to α-AAA-B (12). Even when the concentration of α-AAA-A (3.125 μM) used was half of the concentration of α-AAA-B (6.26 μM), maximum cytotoxicity was exhibited for HL-60 cancer cell line. Moreover, at 6.26 μM concentration of α-AAA-A, which was half of the concentration of α-AAA-B (12.5 μM), maximum cytotoxicity was observed for K-562 cancer cell line. The reason behind higher toxicity of analog A as compared to the analog B is due to a minor structural difference. The acetylenic moiety, which is present on α-AAA-A may have role in the higher potency of the molecule, as compared to the molecule α-AAA-B which retain argylic analog. Removal of the acetylene moiety from an amide derivative (RS)-N-(2-(Benzylamino)-2-oxo-1-phenylethyl)-N-phenylpropiolamide) results in more than 30-fold decrease in potency (12). However, the complete mechanisms behind the cytotoxicities associated with these molecules are still unknown and will be elucidated in future studies. A focused library of biologically active anticancer molecules, which are stable, efficient, cost effective and target various cancers, would be beneficial for efficient therapeutic screening purposes.

Enormous progress has been made recently in adoptive cell therapy treatments for advanced stage cancers. Immune cells have the ability to recognize and remove infected and cancerous cells. Many different types of immune cells, primarily T cells, NK cells and a specialized subset of T cells known as γδ T cells, are some of the candidates, which have been utilized (29–31). Consequently, various technologies focus on boosting function of immune cells by adding agents, aiming to improve their anti-tumor performance. These constitute personalized cancer immunotherapies. Immunotherapies use *in vitro* expanded immune effector cells, which on transference into cancer patients, target tumor cells or stimulate immune response to eliminate them (32). Currently, γδ T cells are an attractive candidate for cancer immunotherapy. We used these cells in this study as they are easy to manipulate *in vitro* and can grow to substantial numbers. These cells can recognize phosphoantigens, such as isopentenyl pyrophosphate produced by stressed cells, as well as bisphosphonates, such as zoledronic acid (14). In this study, expanded γδ T cells exhibited significant *in vitro* killing of both cancer cell lines (HL-60 and K562) at a target to effector ratio of 1:25. We did not further elucidate the differences in killing of two different cell lines. In our previous study, we have also shown the cytotoxic effects of these cells against another chronic myeloid leukemia cell line, i.e., KBM7 (14). Previously, it was observed that expanded γδ T cells retained tumor cell-killing activity without the need for prior activation. However, the myeloid KBM7 cells were much more efficiently killed following overnight incubation with HMBPP. This effect was reduced to the level of untreated KBM7 cells when γδ-TCR blocking Abs were included, demonstrating that the HMBPP pre-treatment of tumor cells directly promoted the γδ-TCR-mediated KBM7 killing. γδ-TCR-blocking Abs did not affect the killing of untreated KBM7 cells, whereas the addition of CD18 and/or NKG2D-blocking Abs reduced the killing of both untreated and HMBPP-pre-treated KBM7 cells.

Similarly, we expect that the mechanism which involve CD18 and/or NKG2D receptors on the expanded γδ T cells, possibly recognize and kill leukemia cancer cells (HL-60 and K562). However, direct blocking assays were not carried out in this study and would be included in future studies to elucidate the mechanism underlying cytotoxicity.

Other *in vitro* studies also showed that γδ T cells kill breast cancer cell lines MDA-MB231, MCF-7, and T47D (33–35). In one of the studies, γδ T cells, in the context of breast cancer, suggested that surface levels of MICA/B on breast cancer cells enhanced targeting and cytotoxicity by γδ T cells against these cell lines (35). Furthermore, the involvement of NKG2D on γδ T cells and MICA/B on MCF-7 and T47D was found in cytotoxicity of γδ T cell against breast tumor targets (36).

Furthermore, γδ T cells have the ability to kill many other tumors (lymphoma, myeloma, melanoma, colorectal, colon, breast, ovary, and prostate cancers) (37).

Pathways for cancer cells are difficult to understand, often due to the involvement of multiple complex molecules. Use of single drug or vaccine poses limitations in countering the complex pathogenesis of cancer. In light of these difficulties, combinational therapies present a unique approach and may provide effective outcomes for many different cancers (9, 24). Importantly, both γδ T cells and the biologically active molecules (α-AAA-A and α-AAA-B), used in this study, are easily produced under *in vitro* conditions and both can be generated in large numbers or amounts. Therefore, both biologically active α-amino amide analogs and γδ T cells are ideal candidates for their use in cancer therapy as combinational therapeutics.

Many studies conducted so far have successfully demonstrated the use of immune cells (T cells, CAR-T cells, NK cells, etc) with small biological molecules [anti-CTLA-4 Abs (Ipilimumab), anti-PD-1 Abs (Nivolumab)] which are immune check point inhibitors (38). Similarly, the combination of amide analogs (α-AAA-A or α-AAA-B) in combination with γδ T cells produced significant killing of two different leukemia cancer cell lines (HL-60 and K562). The killing of cancer cells was markedly substantial when the ratio of cancer cells to γδ T cell was 1:25 and the duration of incubation was 12 h for both molecules. We have discussed earlier the possible mechanisms behind the cytotoxicity of both leukemia cancer cell lines (HL-60 and K562) due to both amide analogs, as well as γδ T cells in combination therapy. Both amide derivative analogs contain acetylene moiety, which has an important role in anticancer activity. Moreover, argylic moiety, which is present on analog A leads to higher potency in combination with γδ T cells as compared with analog-B in combination with γδ T cells. Expanded γδ T cells may show tumor cell killing through the involvement of CD18 and/or NKG2D receptors, which mediate recognition and killing of leukemia cancer cells (HL-60 and K562). Moreover, this form of therapy will not be restricted to a particular type of cancer as most human cancers arouse T-cell responses.

Toxicity and increase of multidrug resistance in cancer patients is a major constraint in chemotherapy (9). Hence, combinational therapeutics have the potential to overcome molecular heterogeneity in patients diagnosed with various cancers. The effect of combinational therapeutics (γδ T cells in combination with biologically active anti-cancer molecules) is better as compared with monotherapies alone. However, it is important to check the toxicities of combinational therapeutics before administration. Preclinical studies are crucial and should be conducted in a regulated manner before clinical trials.

## Conclusions

High yields of novel biologically active molecules (α-AAA-A and α-AAA-B) were achieved with simple reactions, minimum efforts and without any purification. These molecules exhibited cytotoxic activities against leukemia cancer cell lines and remain stable at room temperature. Biologically active molecules can reach a wide spectrum of molecular targets, including intracellular targets or those present deep in the tumor micro-environment. Human γδ T cells exhibit tumor killing activity. The combination of α-AAA-A or α-AAA-B with γδ T cells effectively killed HL-60 and K562 cancer cells in *in vitro* conditions. Thus, biologically active molecule (α-AAA-A and α-AAA-B) and γδ T cells are potential agents for combinational therapy for leukemia. In the future, anticancer molecules may be engineered to perform dual function; first to exhibit cancer cell killing and second to activate *in vivo* γδ T cells.

## Data Availability Statement

The original contributions presented in the study are included in the article/**Supplementary Material**. Further inquiries can be directed to the corresponding author.

## Ethics Statement

This study has been reviewed and approved by the Research Ethics Committee (REC) at the University of Hail dated: 27/11/2020 and approved by university president letter number Nr. 20455/5/42 dated 16/04/1442 H.

## Author Contributions

AO, SS, and MK designed the study. AO, SS, WK, SK, and MK performed the study. AO, SS, MK, SA, EA, and AA wrote the manuscript. All authors contributed to the article and approved the submitted version.

## Funding

This research has been funded by Research Deanship of University of Ha’il, Saudi Arabia through project number RG-191332.

## Conflict of Interest

The authors declare that the research was conducted in the absence of any commercial or financial relationships that could be construed as a potential conflict of interest.
