# Supplementary material for: Biologically Active α-Amino Amide Analogs and γδ T Cells—A Unique Anticancer Approach for Leukemia
Source: Front Oncol. 2021 Jul 12;11:706586. doi: 10.3389/fonc.2021.706586 (PMC8311656; doi:10.3389/fonc.2021.706586)
Supplement: Supplementary file 1 [file DataSheet_1.pdf]

# *Supplementary Material*

## 1.1 Supplementary Figures

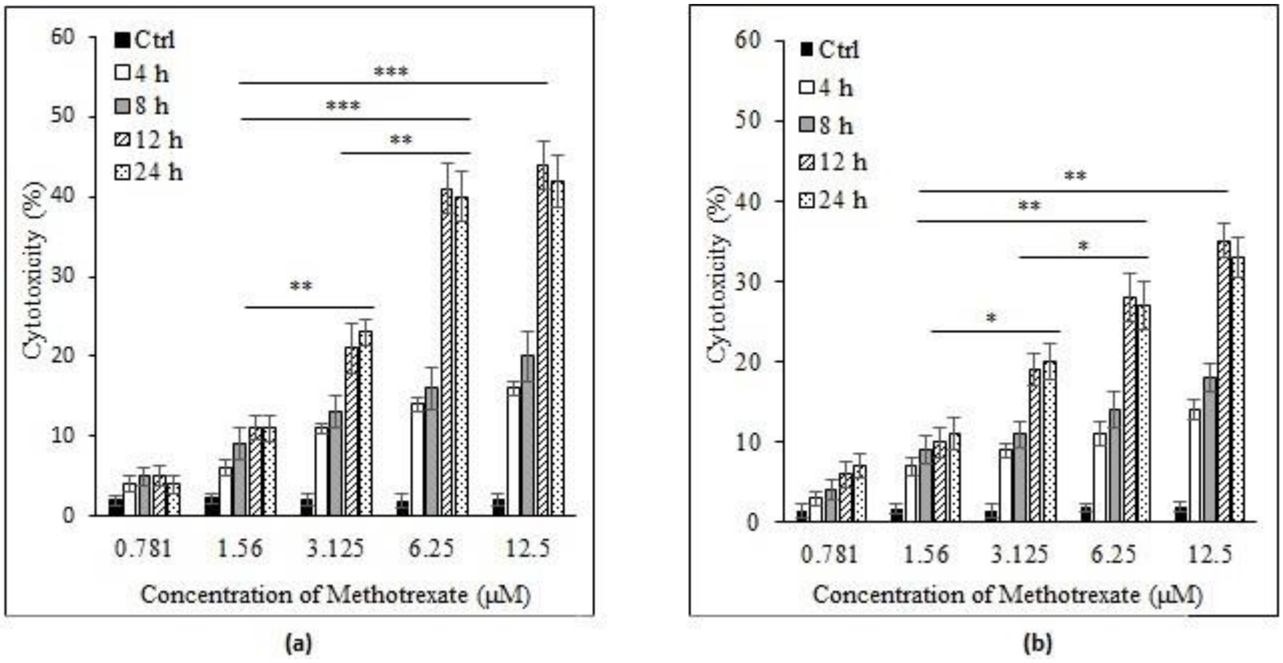


**Supplementary Figure 1.** Cancer cell line HL-60 (a) and K562 (b) cytotoxicities by methotrexate at different concentrations and time durations of incubations. ***$P < 0.001$, on comparing cytotoxicity between of HL-60 cells at 12.5 and 6.25 with 1.56 µM after 12 and 24 h of incubations. **$P < 0.01$, on comparing cytotoxicity of K562 cells at 12.5 and 6.25 with 1.56 µM after 12 and 24 h of incubations. **$P < 0.01$, on comparing cytotoxicity of HL-60 cells between 1.56 and 3.125; between 3.125 and 6.25 µM. *$P < 0.05$, on comparing cytotoxicity of K562 cells at 1.56 and 3.125; between 3.125 and 6.25 µM.

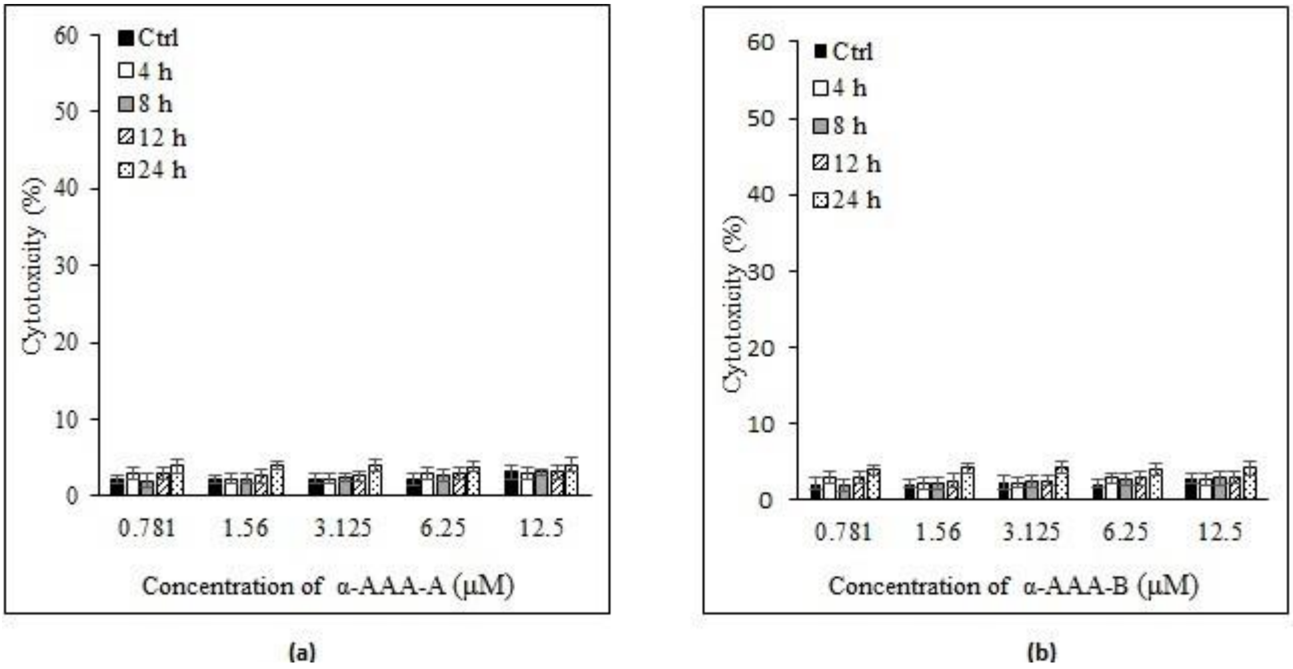


**Supplementary Figure 2.** Fourteen days expanded human γδ T cell cytotoxicities by α-amino amide analogues α-AAA-A (a) and α-AAA-B (b) at different concentrations (0.781 – 12.5 μM) and durations (4 – 24 h) of incubations.

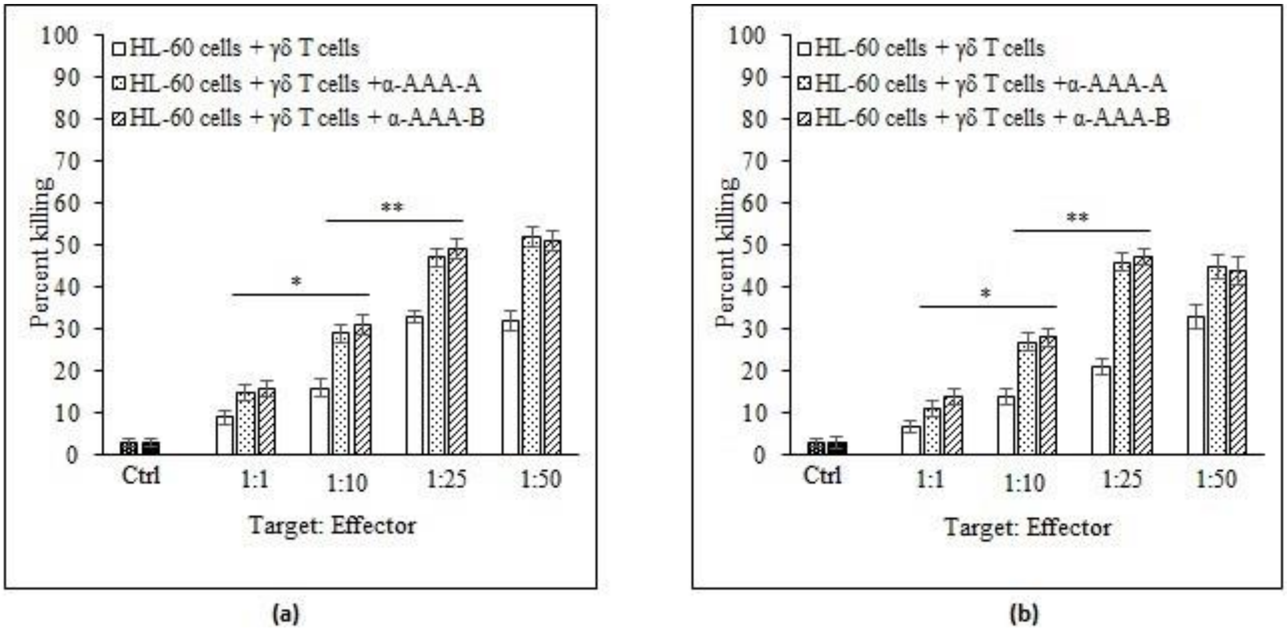


**Supplementary Figure 3.** Combinational effect of methotrexate and expanded γδ T cells. Percent killing of HL-60 (a) and K562 (b) cancer cells by 14 day expanded γδ T cells in combination with methotrexate (12.5 μM). Different T:E ratios were incubated together for a duration of 12h. Cancer cells alone and γδ T cells alone served as controls (ctrl). $**P < 0.01$, on comparison of T:E ratio of 1:25 to 1:10 for both HL-60 and K562 cell. $*P < 0.05$, on comparison of T:E ratio of 1:10 to 1:1 for both HL-60 and K562 cell.
